# Systemic Autoimmunity and Lymphoproliferation Are Associated with Excess IL-7 and Inhibited by IL-7Rα Blockade

**DOI:** 10.1371/journal.pone.0027528

**Published:** 2011-11-10

**Authors:** Rosana Gonzalez-Quintial, Brian R. Lawson, John C. Scatizzi, Joseph Craft, Dwight H. Kono, Roberto Baccala, Argyrios N. Theofilopoulos

**Affiliations:** 1 Department of Immunology and Microbial Science, The Scripps Research Institute, La Jolla, California, United States of America; 2 Department of Immunobiology and Department of Internal Medicine, Yale School of Medicine, New Haven, Connecticut, United States of America; McGill University, Canada

## Abstract

Lupus is characterized by disturbances in lymphocyte homeostasis, as demonstrated by the marked accumulation of activated/memory T cells. Here, we provide evidence that proliferation of the CD8^+^ precursors for the accumulating CD4^–^CD8^–^ T cells in MRL-*Fas^lpr^* lupus-predisposed mice is, in part, driven by commensal antigens. The ensuing lymphadenopathy is associated with increased production of IL-7 due to expansion of fibroblastic reticular cells, the primary source of this cytokine. The excess IL-7 is not, however, consumed by CD4^–^CD8^–^ T cells due to permanent down-regulation of IL-7Rα (CD127), but instead supports proliferation of autoreactive T cells and progression of autoimmunity. Accordingly, IL-7R blockade reduced T cell activation and autoimmune manifestations even when applied at advanced disease stage. These findings indicate that an imbalance favoring production over consumption of IL-7 may contribute to systemic autoimmunity, and correction of this imbalance may be a novel therapeutic approach in lymphoproliferative and autoimmune syndromes.

## Introduction

Lupus, the archetypal systemic autoimmune disease, is characterized by a broad array of T and B cell abnormalities and a plethora of autoantibodies, among which those directed against nucleosomal (DNA, histones) and spliceosomal (small nuclear ribonucleoproteins, snRNP) antigens predominate. Consequent to chronic immune system activation and the effects of various autoantibodies, lupus is characterized by an expedited accumulation of activated T cells as well as lymphopenia, both implying severe disturbances in lymphocyte homeostasis. These disturbances may be attributed to a constellation of factors, including the continuous T cell activation by the ever-present self-antigens, defective activation-induced cell death, and excess of cytokines that promote T cell activation and/or survival. The contribution of these factors may depend on the genetic defect(s) that underlies the pathogenic predisposition. These issues can best be addressed in murine strains that spontaneously develop lupus-like systemic autoimmunity. Among these, the MRL-*Fas^lpr^* model exhibits the most evident disturbance in lymphocyte homeostasis due to an early retroviral transposon insertion in the gene encoding the apoptosis-mediating Fas protein, resulting in defective activation-induced cell death and accumulation of activated/memory T cells.

Recent studies have considerably advanced our understanding of the mechanims by which normal T cell homeostasis is controlled, with two cytokines, IL-7 and IL-15, playing primary roles [1], [2]. IL-7 is mostly produced by fibroblastic reticular cells (FRCs), a mesenchymal cell population found in the stromal environment of lymphoid organs [3], [4]. Binding of IL-7 to the IL-7 receptor (IL-7R), composed of the IL-7Rα chain (CD127) and the common cytokine γ chain (γc, CD132), activates several signaling pathways that enhance cellular metabolic functions and survival of naïve, early effector and memory CD4^+^ and CD8^+^ T cells, primarily by inducing anti-apoptotic Bcl-2 family members [5]. Similarly, IL-15, primarily expressed by activated monocytes and dendritic cells, binds to IL-15Rα (CD359) on accessory cells and is trans-presented to T cells expressing a functional IL-15R, composed of IL-2/15Rβ (CD122) and γc chains [6]. IL-15 promotes the long-term survival of memory CD8^+^ T cells and, in part, naïve CD8^+^ and memory CD4^+^ T cells, but cannot fully compensate for the requirement of IL-7 [1].

In contrast to physiologic conditions in which the availability of IL-7 and IL-15 is rather limited, surplus of these cytokines caused by either reduced consumption (as in lymphopenic mice) or increased production (as in transgenic mice) induces a self-MHC/peptide-dependent T cell expansion, referred to as “homeostatic proliferation”, that ceases only when the equilibrium between cytokine levels and T cell numbers is reestablished [1], [2]. Although largely polyclonal, homeostatic proliferation appears to favor expansion of T cell clones with higher affinity for self-peptides [1], [7], [8], as well as acquisition of several surface markers associated with conventional antigen-induced activation [9], [10] and even effector functions [11]–[13]. We and others, therefore, proposed that continuous or recurrent lymphopenia and the associated cytokine excess may promote the preferential activation and expansion of self-reactive T cells and autoimmunity in predisposed individuals [14]–[17].

In addition to slow-paced homeostatic proliferation occurring under conditions of acute lymphopenia, a second fast-paced form of proliferation termed “spontaneous proliferation” has been observed upon T cell transfer in mice that are chronically lymphopenic due to the absence of recombination activating genes (RAG1 or RAG2) or T cell receptor-encoding genes (TCRα or TCRβ) [18]. In contrast to homeostatic proliferation, spontaneous proliferation does not require IL-7 and is likely driven by commensal rather than self-antigens since it is significantly reduced in germ-free recipients [19]-[21].

Here, we report that both spontaneous and homeostatic proliferation coexist in the MRL-*Fas^lpr^* lupus model. The origin of this homeostasis disturbance could be attributed to self and commensal antigen-induced lymphadenopathy, resulting in expansion of IL-7-producing FRCs and down-regulation of IL-7R in chronically activated T cells. These changes culminate in an excess of IL-7 that sustains the autoimmune process and, thus, blockade of IL-7R signaling significantly decreased disease manifestations in this model.

## Results

### A Subset of MRL-Fas^lpr^ T Cells Exhibit Phenotypic Markers of Homeostatic Proliferation

**Figure 1:**
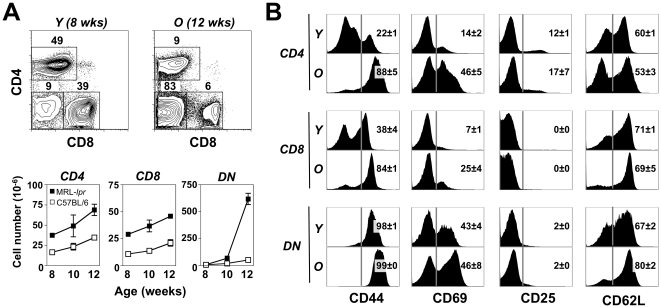
Lymphoaccumulation and T cell phenotype in MRL-*Fas^lpr^* mice. (A) T cell accumulation. LN cells were obtained from MRL-*Fas^lpr^* and control C57BL/6 mice at the indicated ages. Frequency and number (± SD) of CD4^+^, CD8^+^ and DN T cells were determined by flow cytometry after gating on the TCRβ^+^ cell population. (B) T cell phenotype. Expression of CD44, CD69, CD25 and CD62L by TCRβ^+^CD4^+^, TCRβ^+^CD8^+^, and TCRβ^+^DN T cells was assessed by flow cytometry in LNs of young (Y, 8 weeks of age) and older (O, 12 weeks of age) MRL-*Fas^lpr^* mice. Numbers indicate percentage of positive cells (± SD). Data are representative of 3–10 independent experiments with 3–5 mice/group.

In autoimmunity, T cell activation might be induced by either conventional antigen-mediated engagement or excess of T cell trophic cytokines and homeostatic proliferation. These two types of activation can be distinguished by differences in phenotypic cell surface markers, as cognate antigen-activated T cells are CD44^hi^CD69^+^CD25^+^CD62L^low^, whereas homeostatically-expanded T cells are CD44^hi^CD69^–^CD25^–^CD62L^hi^ [9], [22]. As previously described [23], with age and disease progression, MRL-*Fas^lpr^* mice exhibited a moderate expansion of lymph node (LN) CD4^+^ and CD8^+^ single-positive (SP) T cells, and a much larger expansion of CD4^–^CD8^–^ double-negative (DN) T cells (Figure 1A). Interestingly, substantial fractions of SP and DN T cells expressed high levels of CD44 and CD62L, but lacked expression of CD25 and CD69, suggesting that their expansion was caused by excess of T cell-trophic cytokines (Figure 1B). This interpretation is further supported by the finding that most CD25^+^ T cells (99.1±0.1% in young and 96.5±0.9% in old MRL-*Fas^lpr^* mice) were CD4^+^Foxp3^+^ regulatory T cells. However, ∼50% of CD4^+^ and DN T cells, and ∼25% of CD8^+^ T cells in LNs of older mice were CD69^hi^, indicating a concurrent conventional self or foreign antigen-driven activation. These findings suggest that both conventional antigen-mediated engagement and homeostatic proliferation contribute to T cell activation in MRL-*Fas^lpr^* mice.

### DN T Cells Lack Receptors for IL-7 and IL-15

**Figure 2:**
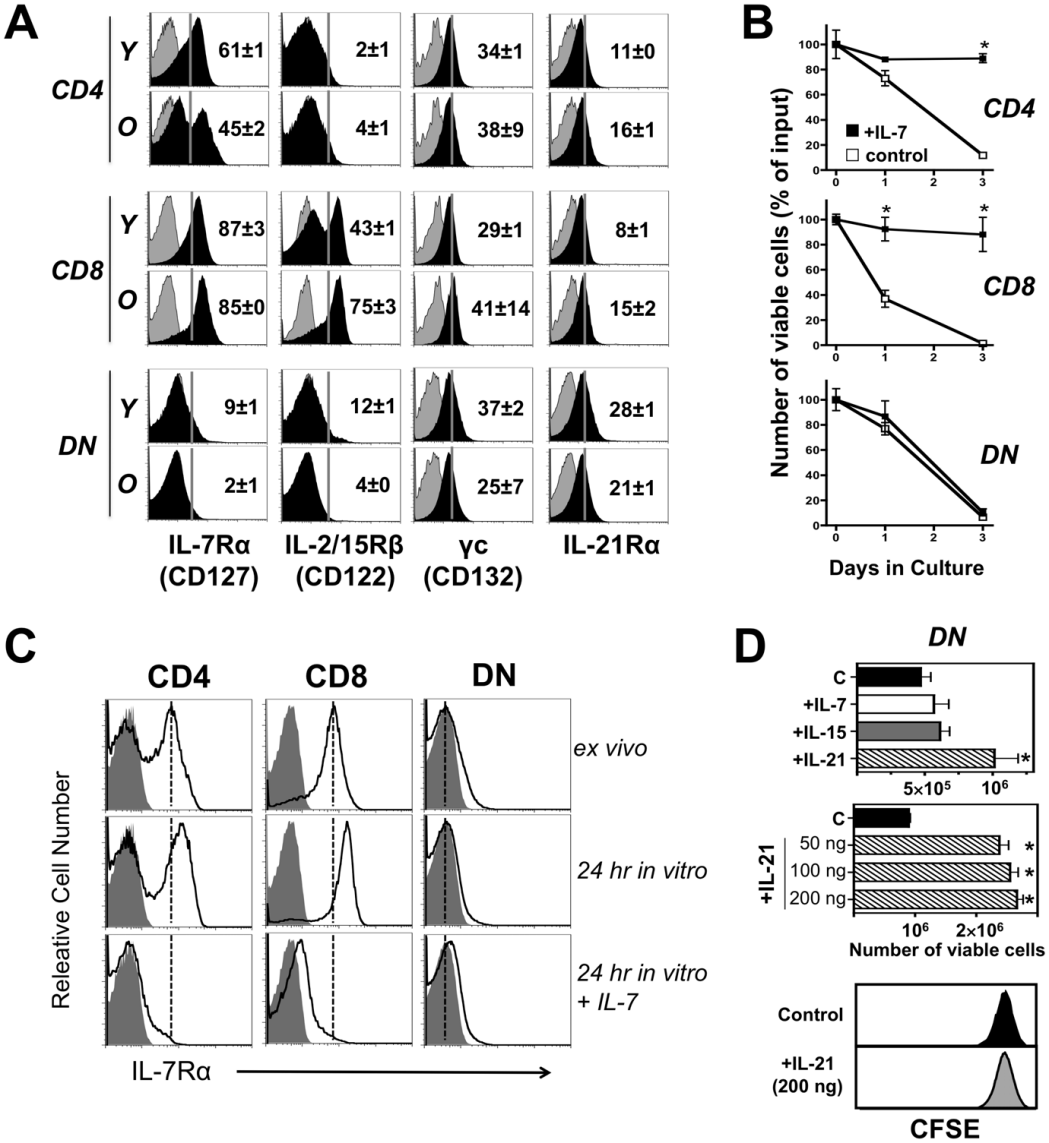
Cytokine receptor expression and survival of MRL-*Fas^lpr^* T cells. (A) IL-7Rα and IL-2/15Rβ down-regulation in DN T cells. Lymph node T cells from young (8 weeks of age) and older (12 weeks) MRL-*Fas^lpr^* mice were analyzed for expression of IL-7Rα (CD127), IL-15Rβ (CD122), γc (CD132) and IL-21R. Numbers indicate percentage of positive cells (± SD). Similar profiles were obtained with spleen cells. (B) Effect of IL-7 on T cell survival. Aliquots (5 × 10^6^) of LN cells were cultured with or without IL-7 (20 ng/ml), and numbers (± SD) of viable CD4^+^, CD8^+^ and DN T cells were determined at the indicated time-points by cell counting upon Trypan Blue staining and flow cytometry. (C) Permanent IL-7Rα down-regulation in DN T cells. LN cells (5 × 10^6^) from MRL*-Fas^lpr^* mice (20 weeks of age) were analyzed either ex vivo, or after 24 hr in vitro culture in the presence of absence of IL-7 (20 ng/ml). (D) Effect of IL-21 on DN T cell survival. DN T cells (5×10^6^) were purified from LNs and cultured for 3 days in medium (control, C), or in the presence of IL-7 (20 ng/ml), IL-15 (10 ng/ml) or IL-21 (50 ng/ml, upper panel; 50-200 ng/ml, middle panel). In addition, DN T cells were CFSE-labeled, cultured with or without IL-21 (200 ng/ml), and proliferation measured by flow cytometry (lower panel). Data are representative of 2-3 independent experiments with 2–5 mice/group. Asterisks indicate statistical significance (p<0.05).

Because T cell survival and homeostatic proliferation are dependent on signaling by IL-7 and IL-15, we examined the expression profiles of the receptors for these cytokines. Depending on age, 45–61% of CD4^+^ T cells and 85–87% of CD8^+^ T cells in spleen and LNs expressed high levels of IL-7Rα, while 43–75% of CD8^+^ T cells, but only 2–4% CD4^+^ T cells, expressed IL-2/15Rβ (Figure 2A), frequencies similar to those in normal mice. Strikingly, however, virtually all DN T cells lacked both these receptors, particularly at advanced age (Figure 2A). Accordingly, in vitro survival of CD4^+^ and CD8^+^ T cells was markedly enhanced by IL-7, whereas DN T cells were unaffected by this cytokine (Figure 2B). Moreover, as previously reported for normal T cells [24], IL-7Rα was down-regulated on both CD4^+^ and CD8^+^ T cells cultured in the presence of IL-7 and up-regulated in the absence of this cytokine, but receptor expression remained undetectable on DN T cells in either condition (Figure 2C), suggesting that IL-7Rα is irreversibly down-regulated on DN T cells. Since most DN T cells are thought to derive from CD8^+^ precursors [25]-[31], we next examined the effects of IL-15 and IL-21, two cytokines known to support survival and proliferation of naïve and memory CD8^+^ T cells [6], [32]. Consistent with IL-2/15Rβ expression, IL-15 enhanced the in vitro survival of CD8^+^, but not DN, T cells. In contrast, IL-21 significantly enhanced survival of DN T cells (Figure 2D, upper and middle panels) without inducing proliferation (Figure 2D, lower panel), an effect that correlated with expression of both IL-21R chains (α and γc) by these cells (Figure 2A). These results indicate that DN T cells lose IL-7Rα and IL-2/15Rβ, cease using IL-7 and IL-15, and switch to alternative survival resources, such as IL-21.

### Commensal Antigens May Contribute to Conversion of CD8^+^ Precursors to DN T Cells

**Figure 3:**
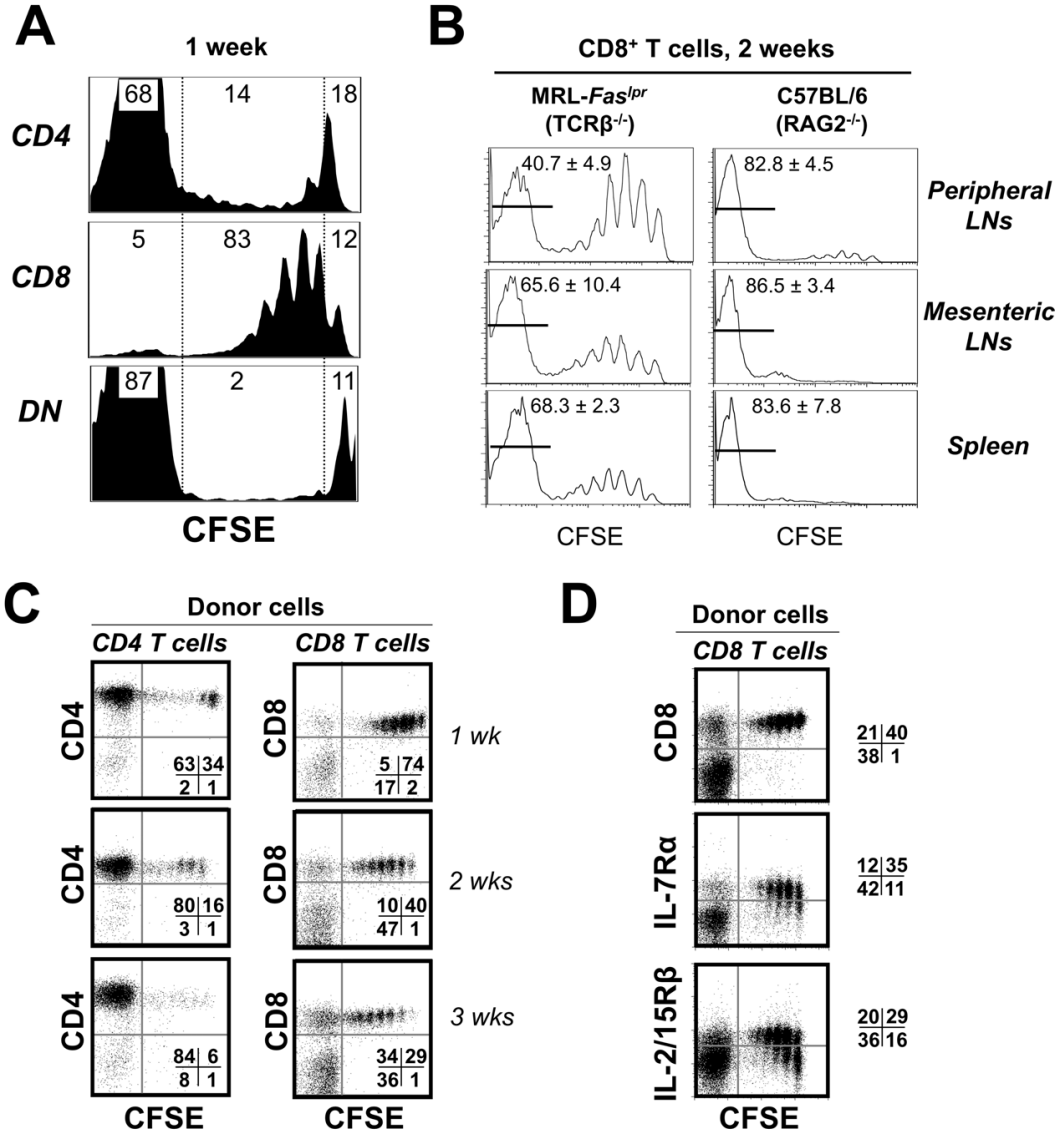
MRL-*Fas^lpr^* T cell proliferation and conversion to DN T cells in T cell-deficient recipients. (A) Homeostatic and spontaneous proliferation. Purified CD4^+^, CD8^+^, and DN T cells from LNs of 12 week-old MRL-*Fas^lpr^* mice were labeled with CFSE and transfused to TCRβ^–/–^ recipients. After 7 days, TCRβ^+^ T cell subsets were harvested from the recipients’ peripheral LNs. Numbers indicate percentage of cells in 0 divisions (CFSE-high), 1–6 divisions (CFSE-intermediate, mostly homeostatic proliferation), and >7 divisions (CFSE-negative, mostly spontaneous proliferation). (B) Reduced spontaneous proliferation of CD8^+^ T cells in chronically immunodeficient TCRβ^–/–^ MRL*-Fas^lpr^* recipients. Total LN cells from MRL-*Fas^lpr^* mice were labeled with CFSE, transferred to TCRβ^–/–^ recipients, and CFSE profiles examined 13 days post-transfer in peripheral LNs, mesenteric LNs and spleen. As controls, LN cells from C57BL/6 mice were CFSE-labeled and transferred to RAG2^–/–^ C57BL/6 recipients. (C) Conversion of CD8^+^ T cells to DN T cells during spontaneous proliferation. Purified CD4^+^ (left panel) and CD8^+^ (right panel) LN T cells were CFSE-labeled and transfused to TCRβ^–/–^ hosts. Gated TCRβ^+^CD8^–^ (left panel) and TCRβ^+^CD4^–^ (right panel) cells were analyzed 1, 2 or 3 weeks post-transfer for the expression of the CD4 or CD8 coreceptors, respectively. Numbers represent percentages of T cells that had either retained (0-6 divisions, upper right quadrant; >7 divisions, upper left quadrant), or lost the coreceptor (0-6 divisions, lower right quadrant; >7 divisions, lower left quadrant). Conversion to DN cells (coreceptor loss) mostly occurred for donor CD8^+^ T cells (right panel) after strong proliferation (i.e., of all the cells that had undergone >7 divisions, 77% had lost CD8 expression at 1 week, 82% at 2 weeks, and 51% at 3 weeks). (D) Conversion to DN T cells during spontaneous proliferation is accompanied by down-regulation of IL-7Rα and IL-2/15Rβ. Purified CD8^+^ T cells from LNs were CFSE-labeled, transfused into TCRβ^–/–^ hosts, and 12 days post-transfer, gated TCRβ^+^CD4^–^ cells were analyzed for the expression of CD8, IL-7Rα and IL-2/15Rβ. Data are representative of 2-3 independent experiments with 3-4 mice/group.

Persistent down-regulation of IL-7Rα and IL-2/15Rβ has been observed on T cells undergoing extensive activation and reaching a functionally defined “exhausted” state [33], [34]. Although accumulation of DN T cells in MRL-*Fas^lpr^* mice is due to defective Fas-mediated apoptosis, the nature of the antigens (self or foreign) that drive activation of the CD8^+^ precursors for DN cells has not been defined. To differentiate between self and foreign stimuli, experiments were performed with purified, LN-derived T cell subsets transferred into TCRβ^–/–^ MRL-*Fas^lpr^* recipients. In these chronically immunodeficient hosts, T cells with slow division, defined as “homeostatic proliferation”, are thought to respond to self-peptide/MHC ligands, whereas those with rapid division, defined as “spontaneous proliferation”, are thought to proliferate in an IL-7-independent manner [18], likely in response to microbiota-derived ligands [19]–[21]. As observed in chronically T cell-deficient normal background mice, at 1 week post-transfer of syngeneic T cells into TCRβ^-/-^ MRL-*Fas^lpr^* recipients, ∼14% of CD4^+^ T cells recovered from peripheral LNs (a pool of inguinal, brachial and axillary) had undergone slow-paced homeostatic proliferation (1 to 7 divisions) and ∼68% fast-paced spontaneous proliferation (>7 divisions) (Figure 3A). Remarkably, however, the vast majority of CD8^+^ T cells (83%) exhibited homeostatic proliferation, whereas DN T cells showed the opposite, i.e. almost exclusively spontaneous proliferation (Figure 3A). At 2 weeks post-transfer, spontaneous proliferation of CD8^+^ T cells was more evident, particularly in mesenteric LNs, the primary site of commensal antigen recognition [19], [20], and in spleen, but remained significantly lower than spontaneous proliferation of control CD8^+^ T cells from C57BL/6 mice transferred into chronically immunodeficient (RAG2^–/–^) syngeneic recipients (Figure 3B).

The reduced spontaneous proliferation of MRL-*Fas^lpr^* CD8^+^ T cells was not due to lack of CD4^+^ T cells or competition with other cell types such as NK and γδ T cells, since slow division of CD8^+^ T cells was maintained when total LN cells were transferred into sublethally-irradiated TCRβ^–/–^ recipients. Instead, reduced spontaneous proliferation could be due to conversion of CD8^+^ T cells to DN cells. Indeed, a significant proportion (∼51–82%) of the CD8^+^ T cells that had undergone >7 divisions in LNs of TCRβ^–/–^ recipients converted to DN T cells (Figure 3C) and down-regulated both IL-7Rα and IL-2/15Rβ (Figure 3D). In contrast, only 3–9% of CD4^+^ T cells that had undergone >7 divisions converted to DN T cells. Interestingly, this conversion was not observed in T cells undergoing slow-paced homeostatic proliferation (Figure 3C and 3D). Hence, the evidence provided by these experiments is compatible with the possibility that recognition of commensal antigens leads to strong proliferation of CD8^+^ T cells, which is associated with down-regulation of both the CD8 coreceptor and the receptors for major T cell trophic cytokines.

### Accumulation of DN T Cells Leads to Excess IL-7

**Figure 4:**
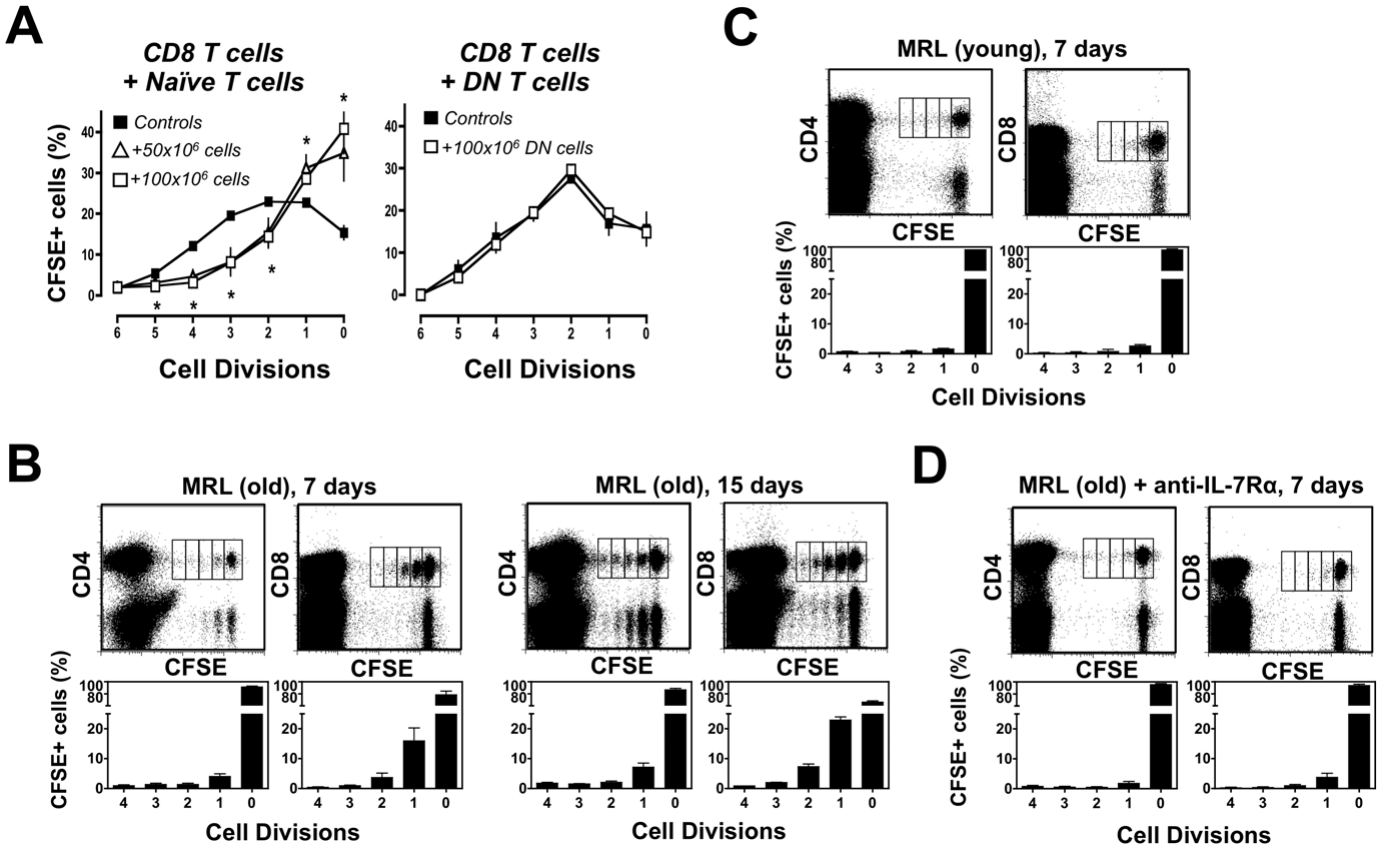
IL-7 excess in old MRL-*Fas^lpr^* mice. (A) DN T cells (IL-7Rα-negative) do not inhibit homeostatic T cell proliferation. CFSE-labeled T cells (3×10^6^) from LNs of young (8 weeks) MRL-*Fas^lpr^* mice were transfused into TCRβ^–/–^ MRL-*Fas^lpr^* recipients alone (control) or together with 50-100 × 10^6^ of either total LN T cells (mostly naïve, lacking DN cells) from young (6 weeks) mice (left panel), or purified DN T cells from older (12 weeks) mice (right panel). Data represent percentages (± SD) of cells in various cell divisions as determined by CFSE profile analysis. (B) T cell proliferation in older non-lymphopenic MRL-*Fas^lpr^* hosts. CFSE-labeled LN T cells (20×10^6^) from older (16 weeks) MRL-*Fas^lpr^* mice were transferred into age-matched unmanipulated (non-lymphopenic) wild-type syngeneic recipients. Seven or 15 days after transfer, LN and spleen cells were harvested and donor CFSE^+^ T cells detected by gating the TCRβ^+^CD4^+^ or TCRβ^+^CD8^+^ cell populations. The frequency (± SD) of donor cells in divisions 0 to 4 was calculated as a percentage of CFSE^+^ cells. (C) No T cell proliferation in young non-lymphopenic MRL-*Fas^lpr^* hosts. The same experiment as in panel B was performed using young (6 weeks) MRL-*Fas^lpr^* mice as T cell donors and non-lymphopenic recipients. (D) T cell proliferation in non-lymphopenic older MRL-*Fas^lpr^* hosts is IL-7-dependent. Older MRL-*Fas^lpr^* mice with lymphadenopathy (16 wks of age) were transfused with CFSE-labeled T cells from age-matched donors and treated with blocking anti-IL-7Rα antibodies three times per week. CFSE proliferation profiles of CD4^+^ and CD8^+^ T cells were examined on day 7 post-transfer. Data are representative of 2–3 independent experiments with 3-4 mice/group.

Down-regulation of IL-7Rα should make DN T cells inefficient consumers of trophic cytokines and therefore unable to inhibit homeostatic proliferation of SP T cells. Moreover, reduced consumption by these cells might create an excess of IL-7 sufficient to provoke proliferation of T cells under non-lymphopenic conditions. Indeed, homeostatic proliferation of CFSE-stained MRL-*Fas^lpr^* SP T cells in LNs of TCRβ^–/–^ recipients was not inhibited by co-transfer of large numbers (100 × 10^6^) of purified DN T cells (Figure 4A). In contrast, efficient inhibition was achieved by co-transfer of total LN cells (50 × 10^6^) from young MRL-*Fas^lpr^* donors, which are mostly composed of naïve T cells with minimal DN T cell expansion (Figure 4A). More importantly, a significant fraction of SP T cells proliferated when transferred into unmanipulated (non-lymphopenic) older MRL-*Fas^lpr^* mice with lymphadenopathy and DN T cell expansion (Figure 4B), whereas no significant proliferation was detected in non-lymphopenic younger MRL-*Fas^lpr^* mice with no lymphadenopathy (Figure 4C). Interestingly, this proliferation was IL-7-dependent and could be inhibited with an anti-IL-7Rα antibody (Figure 4D). These findings indicate that, in parallel with the accumulation of DN T cells lacking IL-7Rα, older MRL-*Fas^lpr^* mice develop excess of IL-7 of sufficient magnitude to induce activation and expansion of potentially autoreactive T cells.

### Lymphadenopathy is Associated with Expansion of IL-7-Producing Stromal Cells

**Figure 5:**
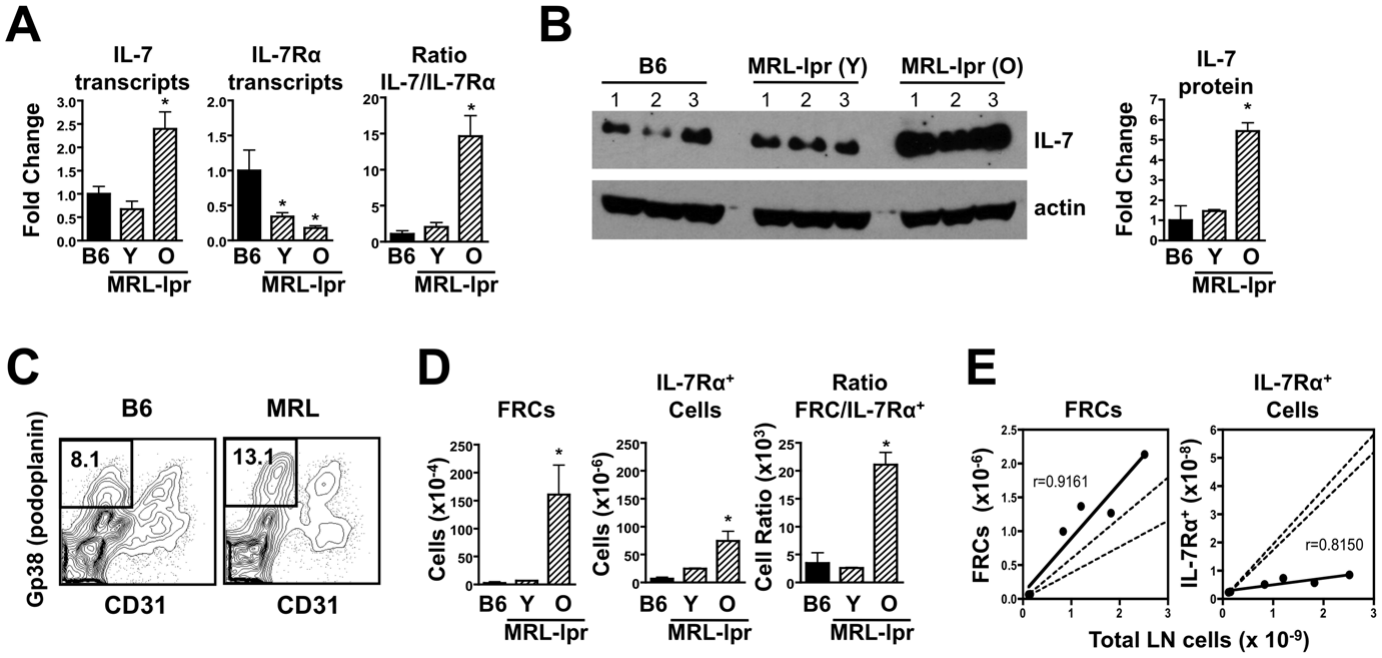
Increased IL-7 production and expansion of fibroblastic reticular cells in LNs of old MRL-*Fas^lpr^* mice. (A) Transcript level analysis. RT-PCR was performed to examine IL-7 and IL-7Rα expression in LNs of young (Y, 6 weeks) and older (O, 20 weeks) MRL-*Fas^lpr^* and control C57BL/6 (B6) mice. Data were normalized using HPRT1 and expressed as fold change (± SD) compared to transcript levels in B6 mice. (B) IL-7 protein levels in young and older MRL-*Fas^lpr^*. Immunoblot was used to determine IL-7 protein levels in LNs of young (Y, 6 weeks) and older (O, 18 weeks) MRL-*Fas^lpr^* and control C57BL/6 (B6) mice. Data acquired by densitometry were normalized using β-actin and expressed as ford change (± SD) compared to IL-7 protein levels in B6 mice. (C) Flow cytometric analysis of fibroblastic reticular cells (FRCs). LN cells of MRL-*Fas^lpr^* and control B6 mice were stained with antibodies to CD45, gp38 (podoplanin) and CD31. Representative plots showing the frequency of FRCs (CD31^–^ gp38^+^) in the CD45^–^ LN cell population are depicted. (D) Accumulation of FRCs and IL-7Rα^+^ T cells in MRL-*Fas^lpr^* mice. The number (± SD) of FRCs and IL-7Rα^+^ T cells in LNs of young and older MRL-*Fas^lpr^* compared to B6 controls was determined by flow cytometry. (E) Accumulation of FRCs is proportional to the number of total LN cells in MRL*-Fas^lpr^* mice. LN cells were isolated from mice displaying various levels of lymphadenopathy. The numbers of FRCs (CD45^–^ CD31^–^ gp38^+^) and IL-7Rα^+^ T cells (TCRβ^+^) were determined by flow cytometry and plotted as a function of the number of total LN cells for each individual mouse. Linear regression and goodness of fit (r) were calculated using Prism 4 software. Dotted lines indicate the frequency ± 1 STD of FRCs (left panel) and IL-7Rα^+^ T cells (right panel) as determined in young mice before development of lymphadenopathy, and predict how these cell types would accumulate with disease progression if their frequencies were maintained at a constant. Data are representative of 2–3 independent experiments with 3–5 mice/group. Asterisks indicate statistical significance (p<0.05).

Because lymphadenopathy may also lead to the expansion of cells that produce IL-7, we examined IL-7 expression as well as frequency of fibroblastic reticular cells (FRCs), the major producers of this cytokine in secondary lymphoid organs [3]. IL-7 transcripts were significantly increased in the enlarged LNs of older MRL-*Fas^lpr^* mice (Figure 5A), while IL-7Rα transcripts were decreased, resulting in ∼7-fold higher ratio of IL-7 to IL-7Rα expression in older compared to younger mice (Figure 5A). Similarly, higher IL-7 protein levels were detected in LNs from older MRL-*Fas^lpr^* mice compared to younger MRL-*Fas^lpr^* mice or C57BL/6 controls (Figure 5B). These findings correlated with an ∼7.6-fold increase in the ratio between the numbers of FRCs and IL-7Rα^+^ T cells (Figure 5C-5D). Furthermore, analysis of MRL-*Fas^lpr^* mice with various degrees of lymphadenopathy indicated that FRCs accumulated proportionally to the number of total LN cells, whereas IL-7Rα^+^ T cells accumulated at much slower rate (Figure 5E). Thus, excess of IL-7 in MRL-*Fas^lpr^* mice is caused by a combination of decreased consumption due to receptor down-regulation by DN T cells, and increased production due to FRC expansion.

### Anti-IL-7Rα Antibody Treatment Reduces Disease in MRL-Fas^lpr^ Mice

**Figure 6:**
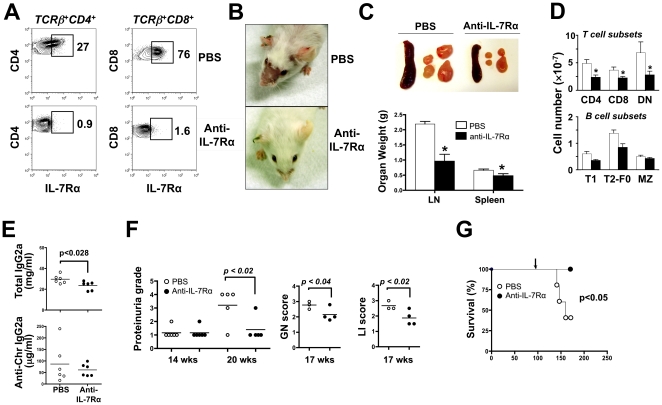
IL-7R blockade inhibits disease in MRL-*Fas^lpr^* mice. (A) IL-7R blockade by anti-IL-7Rα antibody. MRL-*Fas^lpr^* mice (6 weeks old) were treated with anti-IL-7Rα antibody or PBS, 3 times weekly for 4 weeks, and expression of IL-7Rα was determined. (B–E) Prophylactic anti-IL-7Rα antibody treatment. Young female MRL-*Fas^lpr^* mice (6 weeks old) were treated with anti-IL-7Rα or PBS for 6 to 10 weeks. Antibody levels (E) were assessed after 6 weeks, dermatitis (B) after 8 weeks, and weights (± SD) of inguinal, axillary and cervical LNs and spleen (C) after 10 weeks of treatment. B and T cell subsets (D) were examined in LNs and spleen after 10 weeks of treatment. (F-G) Therapeutic anti-IL-7Rα antibody treatment. Female MRL-*Fas^lpr^* mice (14 weeks old) with established disease (lymphadenopathy and anti-chromatin autoantibodies) were treated with anti-IL-7Rα or PBS for 3 to 10 weeks. Proteinuria, glomerulonephritis (GN) and lymphocytic infiltration (LI) were determined between 14 and 20 weeks of age (E), and survival at 24 weeks of age (F). Data are representative of 1–5 independent experiments with 3–9 mice/group.

Since excess of IL-7 signaling may decrease the activation threshold and provoke proliferation of autoreactive T cells, we examined whether blockade of IL-7R could exert therapeutic effects in the MRL-*Fas^lpr^* disease. Initial experiments showed that an anti-IL-7Rα monoclonal antibody (A7R34, rat IgG2a) [35] effectively blocked this receptor in vivo (Figure 6A), and inhibited IL-7-mediated STAT5 phosphorylation in vitro (mean fluorescence intensity for p-STAT5 in CD4^+^ T cells: 295±5 for non-stimulated cells, 587±34 for cells treated with IL-7, and 278±10 for cells treated with IL-7 + anti-IL-7Rα). In vivo, this antibody was effective for up to 4 weeks, with progressive declines thereafter due to immune response against the heterologous antibody. Nonetheless, treatment initiated at early disease stages (6 weeks of age) resulted in significant reductions in dermatitis, lymphadenopathy, splenomegaly and total serum IgG2a, and marginal reduction in anti-chromatin IgG2a autoantibodies, compared to PBS-injected controls (Figure 6B-6E). Treated mice also displayed reduced numbers of CD4^+^, CD8^+^ and DN T cells, as well as immature (T1) and follicular (T2-F0) B cells, whereas marginal zone B cells were unaffected (Figure 6D). The frequency of peritoneal B-1 cells was also unaffected by this treatment (33.1±2.5% vs 26.4±5.3%). Moreover, as expected on the basis of IL-7R expression, there were significant reductions in anti-IL-7Rα-treated vs. control mice at the early developmental stages of bone marrow B cells [pre-/pro-B cells (5.9±0.5% vs. 24.4±1.7%), newly formed B cells (2.1±0.4% vs. 4.5±0.1%) and recirculating B cells (1.6±0.2% vs. 3.8±0.3%)] as well as immature thymocytes [CD44^+^CD25^+^ DN-II cells (3.2±0.3% vs. 6.2±0.1%) and TCRβ^hi^CD69^hi^ cells (6.3±0.8% vs. 9.8±0.2%)].

A separate group of mice was then similarly treated beginning at 14 weeks of age to assess whether IL-7R blockade could inhibit established disease. Indeed, treatment significantly reduced proteinuria, glomerulonephritis, and lymphocyte infiltrates in the kidneys (Figure 6F). Impressively, at 24 weeks of age all antibody-treated mice were alive, compared to >50% mortality in the control group (Figure 6G).

## Discussion

**Figure 7:**
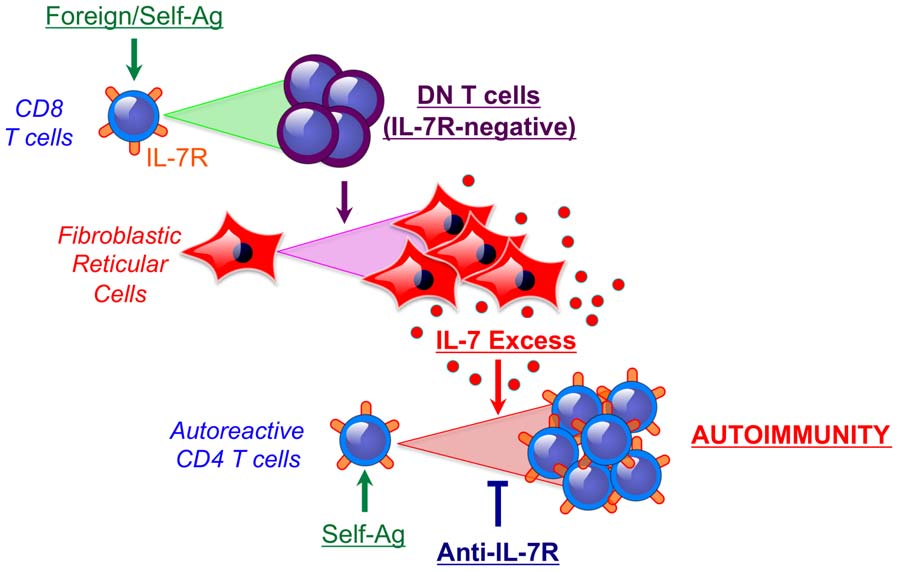
IL-7 excess and systemic autoimmunity in MRL-*Fas^lpr^* mice. The following model is proposed: commensal and self-antigens in MRL-*Fas^lpr^* lupus mice induce proliferation of CD8^+^ T cells and conversion to double-negative (DN) T cells that irriversibly down-regulate IL-7R. Lymphoaccumulation causes reorganization of the lymphoid organ microarchitecture and expansion of the stromal cell compartment, including fibroblastic reticular cells (FRCs), the main source of IL-7. The resulting excess of IL-7 is not used by DN cells, but instead promotes autoreactive T cell activation and proliferation. Consequently, antibody-mediated blockade of IL-7R signaling reduces autoimmunity in this model.

We performed studies to define homeostatic characteristics of T cells in the MRL-*Fas^lpr^* lupus model, focusing on potential imbalances in the production and consumption of the major T cell prosurvival cytokines. We found that a substantial proportion of T cells displayed phenotypic markers resembling those of T cells undergoing homeostatic proliferation. In addition, the DN T cells that massively accumulate in this Fas-defective lupus model were found to permanently down-regulate the receptors for IL-7 and IL-15, probably due to chronic activation in part mediated by commensal antigens, leading to reduced consumption of these cytokines. Lymphoaccumulation in these mice was also associated with expansion of FRCs, increased IL-7 production, and enhanced IL-7-dependent T cell proliferation. Consequently, treatment with an IL-7Rα-blocking antibody significantly reduced autoimmune disease manifestations in this model (Figure 7).

We and others have hypothesized that, in predisposed genetic backgrounds, recurrent homeostatic T cell proliferation triggered by lymphopenia and excess of T cell-trophic cytokines may be a contributing factor in the pathogenesis of systemic autoimmunity [14]-[17]. This possibility stems from the observation that during homeostatic proliferation T cells acquire effector functions and are selected for high affinity to self-antigens [11]-[13]. Indeed, a considerable proportion of T cells in MRL-*Fas^lpr^* mice displayed surface markers comparable to those acquired by normal T cells during homeostatic proliferation, and similar phenotypic characteristics have been reported for C57BL/6-*Fas^lpr^* T cells [36]. Although this phenotype may also be acquired by conventional antigen-induced effectors during conversion to central memory T cells, this seems unlikely since classical T cell activation is associated with transient expression of CD25 [9], [37], yet this marker was primarily detected on Foxp3^+^ regulatory T cells and, at low levels, in a very small population (<5%) of Foxp3^–^ CD4^+^ T cells. Nonetheless, a substantial fraction of CD4^+^, CD8^+^ and DN T cells expressed the CD69 marker, typically upregulated during antigen-induced activation. Thus, both conventional (self) antigens and homeostatic proliferation seem to contribute to T cell activation in this model.

The present study indicates that homeostatic T cell proliferation in these mice is driven by excess of IL-7, since adoptively-transferred SP T cells proliferated in unmanipulated older MRL-*Fas^lpr^* recipients, and this proliferation was significantly blocked by administration of anti-IL-7Rα antibody. Similar observations were reported for C57BL/6-*FasL^gld^* mice, which also exhibit expansion of DN T cells with reduced expression of IL-7Rα and IL-2/15Rβ [38]. Two major mechanisms emerge from the present study that might explain how IL-7 excess is attained in these mice: increased production due to the expansion of FRCs in the enlarged LNs, and decreased consumption due to persistent down-regulation of IL-7Rα by DN T cells.

FRCs constitute a unique type of stromal cells that enwrap conduits, consisting of collagen fibers and other extracellular matrix proteins, that distribute lymph and small molecules throughout the T cell zone of secondary lymphoid organs [4]. FRCs have been identified as the major source of IL-7 and other factors essential for T cell homeostasis and trafficking [3]. Presumably, lymphoaccumulation of DN T cells induces reorganization of the microarchitecture in the enlarged LNs, leading to increased FRC numbers, as was reported to occur during LN organogenesis, or following certain infections that transiently deplete FRC networks [4].

Regarding the second mechanism, transient down-regulation of IL-7Rα has been previously shown in thymocytes during transition from double-negative to double-positive stages [5], and in mature T cells following either TCR engagement or signaling by IL-7 and other pro-survival cytokines [24]. This transient IL-7Rα down-regulation is required for efficient IL-7 signaling [39], but has also been proposed as an “altruistic” mechanism to ensure availability of this survival-promoting cytokine to unstimulated T cells [5]. Contrastingly, for the MRL-*Fas^lpr^* DN T cells, down-regulation of IL-7Rα (and IL-2/15Rβ) appears irreversible. Irreversible down-regulation of IL-7Rα also occurs in functionally “exhausted” T cells that accumulate during persistent viral infections, such as HIV or LCMV [33], [34] and, interestingly, high levels of IL-7 can be detected in the serum of AIDS patients [33]. Therefore, it is likely that the DN T cells of MRL-*Fas^lpr^* mice have also reached an exhaustion state, which is further suggested by an age-related progressive decline in proliferative capacity of these cells both in vitro and in vivo [31], [40]. The molecular mechanism by which IL-7Rα expression is regulated has not been fully explained. Nonetheless, IL-7Rα endocytosis is mediated by clathrin-coated pits followed by Jak3- and proteasome-dependent degradation [39], while resynthesis is regulated by several factors, including the transcriptional repressor growth factor independence-1 (GFI1) and the transcription factor Forkhead box O1 (Foxo1)[24], [41].

Absence of IL-7R and IL-15R in DN T cells raises questions on the nature of alternative resources utilized by these cells for survival. We obtained evidence that this function might, in part, be subserved by IL-21, another prosurvival cytokine for naïve and activated/memory T cells [32]. Interestingly, blockade of IL-21 has been reported to reduce the MRL-*Fas^lpr^* disease, including lymphadenopathy [42], and this cytokine has been shown to support the survival and function of chronically-activated CD8^+^ T cells during a persistent viral infection [43].

The nature of the activating stimulus that promotes conversion of CD8^+^ precursors to DN T cells has not been directly defined. However, based on the present adoptive transfer experiments in *chronically* immunodeficient recipients, we have hypothesized that commensal antigens may in part be responsible for driving this process. Specifically, we found that a) DN T cells exhibited *spontaneous proliferation*, which is attributed to microbiota-derived antigens and DC activation by innate stimuli [19]–[21], but not *homeostatic proliferation*, which is attributed to self-peptide/MHC recognition [1], [2]; b) the CD8^+^ T cell repertoire of MRL-*Fas^lpr^* mice was largely depleted of cells able to undergo spontaneous proliferation, presumably because of their conversion to DN cells; and c) the small CD8^+^ fraction that displayed spontaneous proliferation converted to DN T cells and down-regulated IL-7Rα and IL-2/15Rβ. Previous studies indicated that down-regulation of CD8 may result from sustained antigen stimulation [44], [45], or reduced IL-7 signaling [46], suggesting that down-regulation of IL-7Rα may precede that of CD8. Moreover, the small population of DN T cells present in normal mice was reported to display anti-bacteria specificity [47], and MRL-*Fas^lpr^* mice bred in a germ-free environment and fed an antigen-free diet had significant reductions in lymphadenopathy and other disease characteristics [48]. The findings as a whole suggest that activation of CD8^+^ T cells and conversion to DN cells may, in part, be driven by commensal rather than self-ligands, likely due to defects in the anatomic barriers that limit bacterial translocation [49], and/or defects in the suppressive mechanisms that curtail responses to these ligands [1]. Adoptive transfer experiments, similar to those described in the present study, with germ-free MRL-*Fas^lpr^* recipients will allow more definitive conclusions with regard to the role of commensal antigens in driving CD8^+^ T cell activation and conversion to DN cells in this model.

Studies with *Fas^lpr^* mice lacking MHC class I, MHC class II, CD4, or CD8 suggested that CD8^+^ T cells and the derivative DN cells are not essential for autoimmune disease development, whereas CD4^+^ T cells are required [26]–[31]. Nonetheless, in some of these studies, mice lacking CD8^+^ and DN T cells showed significant reductions in serologic and histologic disease parameters [28]–[31]. The present results suggest that DN T cells contribute to disease indirectly by reducing the cellular “sinks” for IL-7 and promoting expansion of cells that produce this cytokine. Similar changes may occur in SLE and in autoimmune lymphoproliferative syndrome (ALPS), where there is expansion of DN T cells [50]–[52].

The ultimate effect of IL-7 excess in autoimmunity is a reduction of the activation threshold of autoreactive T cells, which, when superimposed on a predisposed genetic background, leads to disease enhancement. Hence, treatment of MRL-*Fas^lpr^* mice with an IL-7Rα-blocking antibody exerted significant disease-decreasing effects even when applied at an advanced disease stage. These effects are compatible with the primary role of IL-7 in survival and proliferation of naïve, early effector, and memory CD4^+^ T cells. It is possible, however, that additional mechanisms may contribute to the therapeutic effects, including reduction of autoreactive B cells, the development of which is dependent on IL-7 in mice [5], [6]. Moreover, IL-7Rα is also a component of the thymic stromal lymphopoietin (TSLP) [53], a cytokine previously shown to promote systemic autoimmunity when overexpressed in transgenic mice [54]. Thus, disease reduction in anti-IL-7Rα antibody-treated mice may be due to inhibition of both IL-7 and TSLP, and additional experiments with specific antibodies or targeted gene deletion may clarify the differential contribution of these cytokines in systemic autoimmunity. Considering that autoimmune disease pathogenesis invariably involves the participation of CD4^+^ T cells, it is likely that blockade of IL-7Rα signaling will be an efficient treatment in a wide spectrum of autoimmune and inflammatory disorders.

## Materials and Methods

### Mice

Animals were housed in a facility approved by the Association for Assessment and Accreditation of Laboratory Animal Care International. All animal experiments and protocols were performed according to the NIH Guide for the Care and Use of Laboratory Animals and approved by The Scripps Research Institute Animal Care Committee (permit numbers 09-0082 and 09-0101). MRL-*Fas^lpr^*, C57BL/6 (B6) and C57BL/6.Rag2^–/–^ mice were obtained from The Jackson Laboratory (Bar Harbor, ME), and T cell-deficient TCRβ^–/–^ MRL-*Fas^lpr^* mice have been previously described [55]. These mice were maintained at the mouse facility of The Scripps Research Institute under specific pathogen-free conditions.

### Cell Preparations

Single cell suspensions were prepared from bone marrow, thymus, spleen and LNs (inguinal, axillary, brachial, cervical and, when indicated, mesenteric). To analyze B cell precursors in bone marrow, cells were stained with antibodies to Igk and CD19, and pre-/pro-B cells (CD19^+^ Igk^–^), newly formed B cells (CD19^low^ Igk^low^) and recirculating B cells (CD19^hi^ Igk^hi^) were identified by flow cytometry. Similarly, flow cytometry was used to enumerate T cell precursors (DN-I to DN-IV, TCRβ^low^CD69^low^, TCRβ^hi^CD69^hi^) upon thymocyte staining with antibodies to CD4, CD8, CD44, CD25, CD69 and TCRβ. To analyze FRCs, LN tissues were digested with collagenase IV and DNase I, and the obtained single cell suspensions were stained with antibodies to CD45, gp38 (podoplanin) and CD31, and FRCs were identified by flow cytometry as CD45^–^CD31^–^ gp38^+^ cells, as described [3]. For some in vivo and in vitro experiments, purified CD4^+^, CD8^+^ and DN T cell subsets were isolated by cell sorting using monoclonal antibodies to mouse CD19 and I-A^k^ (to exclude non-T cells), as well as CD4 and CD8. As assessed by flow cytometry using fluorescent antibodies to TCRβ, CD4 and CD8, purity was typically 96–98%.

### Flow Cytometry

Monoclonal antibodies to mouse TCRβ, CD4, CD8, CD44, CD25, CD69, CD62L, CD127, CD122, CD132, Foxp3, IL-21R, pSTAT5, CD19, Igk, CD23, CD21, CD5, B220, gp38 (podoplanin), CD31 and CD45, and streptavidin (conjugated to FITC, PE, PerCP, or APC) were commercially obtained (BD Pharmingen, Biolegend or eBioscience). For surface staining, cells were sequentially incubated with various combinations of antibodies or streptavidin. For detection of Foxp3, fixed cells were permeabilized and stained with a specific antibody, as recommended by the manufacturer (eBioscience). Cell events were acquired on four-color FACSCalibur™ (BD Pharmingen) and data were analyzed using FlowJo software (Tree Star).

### Real Time Quantitative RT-PCR

LN cells were obtained from young (6 wks) and older (20 wks) MRL-*Fas^lpr^* mice and control C57BL/6 mice (12 wks) (n = 3 mice/group). RNA was extracted from total LN cells (RNeasy Plus Mini kit, Qiagen) and cDNA synthesized (RT First Strand Kit, SABiosciences). PCR was performed in triplicate using 400 ng cDNA, the RT SYBR Green qPCR Master Mix (SABiosciences), primer sets specific for mouse IL-7, IL-7Rα or HPRT1 gene sequences (SABiosciences), and an ABI PRISM 7900HT Sequence Detection System (PE Biosystems). IL-7 and IL-7Rα transcript levels in individual mice were normalized using HPRT1 values and expressed as fold change in comparison to levels in control C57BL/6 (B6) mice.

### Immunoblot

LNs were obtained from young (6 wks) and older (18 wks) MRL-*Fas^lpr^* mice and control C57BL/6 mice (16 wks) (n = 3 mice/group). LNs were minced with a razor blade and lysed overnight at 4°C in M-Per mammalian extraction buffer containing 150 mM NaCl_2_ (Thermo Scientific). Protein concentrations were determined (Bio-Rad Protein Assay) and, for each individual mouse, 75 µg separated on a precast 4–12% gradient polyacrylamide gel (Invitrogen) under reducing conditions. Proteins were transferred to a nitrocellulose membrane using Invitrogen iBlot technology and antibody incubation and washes were performed using an Invotrogen BenchPro 410 card processing station. Briefly, membranes were incubated with rat anti-mouse IL-7 monoclonal antibody (R&D Systems) followed by peroxidase-conjugated goat anti-rat IgG antibody (Jackson ImmunoResearch Labs) and SuperSignal chemiluminescent substrate (Thermo Scientific). After film (ThermoScientific) exposure, nitrocellulose membranes were stripped and re-probed using a rabbit anti-mouse β-actin antibody (Cell Signaling Technology) followed by peroxidase-conjugated donkey anti-rabbit IgG antibody (BioLegend). Films were examined by densitometry (ImageJ software) and, for each individual mouse, band intensities were normalized by subtracting the background and calculating the ratio IL-7/β-actin.

### Adoptive Cell Transfers

Total LN cells or purified CD4^+^, CD8^+^ and DN T cells from MRL-*Fas^lpr^* mice were stained with 5,6-carboxyfluorescein diacetate succinimidyl ester (CFSE, Molecular Probes). Briefly, cells were washed in PBS containing 0.1% BSA (PBS/0.1%BSA), resuspended to 10^7^ cells/ml in pre-warmed (37°C) PBS/0.1%BSA containing 10 µM CFSE, incubated for 10 min at 37°C, and washed once with cold DMEM containing 20% FCS and once with cold DMEM. Aliquots of 3 to 20×10^6^ CFSE-stained cells were injected intravenously (i.v.) into T cell-deficient (TCRβ^–/–^) or lymphosufficient (wild-type) MRL-*Fas^lpr^* recipients. In some experiments, the CFSE-stained cells were co-injected with 50-100×10^6^ of unstained total LN cells or purified DN T cells. At the indicated time points, mice were sacrificed, and LN and spleen cells were analyzed by flow cytometry.

### In Vitro Studies

Total LN cells or purified DN T cells were cultured in complete RPMI-1640 (10% FCS, 2 mM L-glutamine, 20 U/ml penicillin, and 20 µg/ml streptomycin) supplemented with recombinant IL-7 (20 ng/ml), IL-15 (10 ng/ml) or IL-21 (50, 100 or 200 ng/ml). At the indicated time-points, cells were harvested, counted, and the numbers of viable CD4^+^, CD8^+^ and DN T cells were determined on the basis of Trypan Blue exclusion and flow cytometry analysis. To evaluate IL-7 signaling-inhibition by the anti-IL-7Rα antibody (A7R34), splenocytes (5×10^6^) were cultured for 10 min with recombinant IL-7 (100 ng/ml), anti-IL-7Rα (10 µg/ml), combinations of IL-7 and anti-IL-7Rα, or medium alone. Cells were then washed, stained with antibodies to TCRβ and CD4, fixed, permeabilized, intracellularly stained with antibodies to pSTAT5 (Cell Signaling Technology) and analyzed by flow cytometry.

### In Vivo Treatment with Anti-IL-7Rα Antibodies

MRL-*Fas^lpr^* mice were treated i.p. with 200 µg anti-IL-7Rα rat monoclonal antibody (clone A7R34) three times per week for 1 to 10 weeks. Treatment was started either before (6 weeks of age) or after (14 weeks of age) appearance of disease manifestations (i.e. dermatitis, lymphadenopathy and autoantibody titers).

### Serology

Total and anti-chromatin serum IgG subclasses were assessed by ELISA using 96-well plates coated with goat anti-mouse IgG (Jackson ImmunoResearch Laboratories) or mouse chromatin, respectively. Bound antibodies were detected using alkaline phosphatase-conjugated goat antibodies (Caltag Laboratories) to mouse IgG or IgG2a, the main autoantibody subclass in this model. Standard curves were generated using calibrated mouse serum (Accurate Chemical and Scientific Company).

### Kidney Pathology

Levels of proteinuria were determined using reagent strips for urinalysis (Albustix, Bayer Corporation) and graded semiquantitatively (0  =  negative to traces, 1  =  30 mg/ml; 2  =  100 mg/ml, 3  =  300 mg/ml, 4  =  2000 mg/ml). The severity of glomerulonephritis (GN) and lymphocytic infiltrates were scored blindly on a 0–4 scale, as described [56].

### Statistical Analysis

Group comparisons were analyzed by unpaired two-tailed Student’s *t* test. Survival was analyzed by Kaplan-Meier plot and log rank test. *p*<0.05 was considered significant.

## References

[1] Surh CD, Sprent J (2008). Homeostasis of naive and memory T cells.. Immunity.

[2] Takada K, Jameson SC (2009). Naive T cell homeostasis: from awareness of space to a sense of place.. Nat Rev Immunol.

[3] Link A, Vogt TK, Favre S, Britschgi MR, Acha-Orbea H (2007). Fibroblastic reticular cells in lymph nodes regulate the homeostasis of naive T cells.. Nat Immunol.

[4] Junt T, Scandella E, Ludewig B (2008). Form follows function: lymphoid tissue microarchitecture in antimicrobial immune defence.. Nat Rev Immunol.

[5] Mazzucchelli R, Durum SK (2007). Interleukin-7 receptor expression: intelligent design.. Nat Rev Immunol.

[6] Ma A, Koka R, Burkett P (2006). Diverse functions of IL-2, IL-15, and IL-7 in lymphoid homeostasis.. Annu Rev Immunol.

[7] Ge Q, Rao VP, Cho BK, Eisen HN, Chen J (2001). Dependence of lymphopenia-induced T cell proliferation on the abundance of peptide/MHC epitopes and strength of their interaction with T cell receptors.. Proc Natl Acad Sci U S A.

[8] Kassiotis G, Zamoyska R, Stockinger B (2003). Involvement of avidity for major histocompatibility complex in homeostasis of naive and memory T cells.. J Exp Med.

[9] Murali-Krishna K, Ahmed R (2000). Cutting edge: naive T cells masquerading as memory cells.. J Immunol.

[10] Goldrath AW, Luckey CJ, Park R, Benoist C, Mathis D (2004). The molecular program induced in T cells undergoing homeostatic proliferation.. Proc Natl Acad Sci U S A.

[11] Cho BK, Rao VP, Ge Q, Eisen HN, Chen J (2000). Homeostasis-stimulated proliferation drives naive T cells to differentiate directly into memory T cells.. J Exp Med.

[12] Dummer W, Niethammer AG, Baccala R, Lawson BR, Wagner N (2002). T cell homeostatic proliferation elicits effective antitumor autoimmunity.. J Clin Invest.

[13] Hamilton SE, Wolkers MC, Schoenberger SP, Jameson SC (2006). The generation of protective memory-like CD8+ T cells during homeostatic proliferation requires CD4+ T cells.. Nat Immunol.

[14] Theofilopoulos AN, Dummer W, Kono DH (2001). T cell homeostasis and systemic autoimmunity.. J Clin Invest.

[15] Stockinger B, Kassiotis G, Bourgeois C (2004). Homeostasis and T cell regulation.. Curr Opin Immunol.

[16] Baccala R, Theofilopoulos AN (2005). The new paradigm of T-cell homeostatic proliferation-induced autoimmunity.. Trends Immunol.

[17] Krupica T, Fry TJ, Mackall CL (2006). Autoimmunity during lymphopenia: a two-hit model.. Clin Immunol.

[18] Min B, Yamane H, Hu-Li J, Paul WE (2005). Spontaneous and homeostatic proliferation of CD4 T cells are regulated by different mechanisms.. J Immunol.

[19] Kieper WC, Troy A, Burghardt JT, Ramsey C, Lee JY (2005). Recent immune status determines the source of antigens that drive homeostatic T cell expansion.. J Immunol.

[20] Tajima M, Wakita D, Noguchi D, Chamoto K, Yue Z (2008). IL-6-dependent spontaneous proliferation is required for the induction of colitogenic IL-17-producing CD8+ T cells.. J Exp Med.

[21] Feng T, Wang L, Schoeb TR, Elson CO, Cong Y (2010). Microbiota innate stimulation is a prerequisite for T cell spontaneous proliferation and induction of experimental colitis.. J Exp Med.

[22] Goldrath AW, Bogatzki LY, Bevan MJ (2000). Naive T cells transiently acquire a memory-like phenotype during homeostasis-driven proliferation.. J Exp Med.

[23] Theofilopoulos AN, Dixon FJ (1985). Murine models of systemic lupus erythematosus.. Adv Immunol.

[24] Park JH, Yu Q, Erman B, Appelbaum JS, Montoya-Durango D (2004). Suppression of IL7Ralpha transcription by IL-7 and other prosurvival cytokines: a novel mechanism for maximizing IL-7-dependent T cell survival.. Immunity.

[25] Landolfi MM, Van Houten N, Russell JQ, Scollay R, Parnes JR (1993). CD2-CD4-CD8- lymph node T lymphocytes in MRL lpr/lpr mice are derived from a CD2+CD4+CD8+ thymic precursor.. J Immunol.

[26] Jevnikar AM, Grusby MJ, Glimcher LH (1994). Prevention of nephritis in major histocompatibility complex class II-deficient MRL-lpr mice.. J Exp Med.

[27] Koh DR, Ho A, Rahemtulla A, Fung-Leung WP, Griesser H (1995). Murine lupus in MRL/lpr mice lacking CD4 or CD8 T cells.. Eur J Immunol.

[28] Mixter PF, Russell JQ, Durie FH, Budd RC (1995). Decreased CD4-CD8- TCR-alpha beta + cells in lpr/lpr mice lacking beta 2-microglobulin.. J Immunol.

[29] Maldonado MA, Eisenberg RA, Roper E, Cohen PL, Kotzin BL (1995). Greatly reduced lymphoproliferation in lpr mice lacking major histocompatibility complex class I.. J Exp Med.

[30] Christianson GJ, Blankenburg RL, Duffy TM, Panka D, Roths JB (1996). beta2-microglobulin dependence of the lupus-like autoimmune syndrome of MRL-lpr mice.. J Immunol.

[31] Balomenos D, Rumold R, Theofilopoulos AN (1997). The proliferative in vivo activities of lpr double-negative T cells and the primary role of p59fyn in their activation and expansion.. J Immunol.

[32] Leonard WJ, Spolski R (2005). Interleukin-21: a modulator of lymphoid proliferation, apoptosis and differentiation.. Nat Rev Immunol.

[33] Rethi B, Fluur C, Atlas A, Krzyzowska M, Mowafi F (2005). Loss of IL-7Ralpha is associated with CD4 T-cell depletion, high interleukin-7 levels and CD28 down-regulation in HIV infected patients.. Aids.

[34] Lang KS, Recher M, Navarini AA, Harris NL, Lohning M (2005). Inverse correlation between IL-7 receptor expression and CD8 T cell exhaustion during persistent antigen stimulation.. Eur J Immunol.

[35] Sudo T, Nishikawa S, Ohno N, Akiyama N, Tamakoshi M (1993). Expression and function of the interleukin 7 receptor in murine lymphocytes.. Proc Natl Acad Sci U S A.

[36] Fortner KA, Budd RC (2005). The death receptor Fas (CD95/APO-1) mediates the deletion of T lymphocytes undergoing homeostatic proliferation.. J Immunol.

[37] Dooms H, Wolslegel K, Lin P, Abbas AK (2007). Interleukin-2 enhances CD4+ T cell memory by promoting the generation of IL-7R alpha-expressing cells.. J Exp Med.

[38] Aranami T, Iclozan C, Iwabuchi K, Onoe K (2004). IL-7-dependent homeostatic proliferation in the presence of a large number of T cells in gld mice.. Microbiol Immunol.

[39] Henriques CM, Rino J, Nibbs RJ, Graham GJ, Barata JT (2010). IL-7 induces rapid clathrin-mediated internalization and JAK3-dependent degradation of IL-7Ralpha in T cells.. Blood.

[40] Altman A (1994). Abnormal antigen receptor-initiated signal transduction in lpr T lymphocytes.. Semin Immunol.

[41] Kerdiles YM, Beisner DR, Tinoco R, Dejean AS, Castrillon DH (2009). Foxo1 links homing and survival of naive T cells by regulating L-selectin, CCR7 and interleukin 7 receptor.. Nat Immunol.

[42] Herber D, Brown TP, Liang S, Young DA, Collins M (2007). IL-21 has a pathogenic role in a lupus-prone mouse model and its blockade with IL-21R.Fc reduces disease progression.. J Immunol.

[43] Elsaesser H, Sauer K, Brooks DG (2009). IL-21 is required to control chronic viral infection.. Science.

[44] Erard F, Wild MT, Garcia-Sanz JA, Le Gros G (1993). Switch of CD8 T cells to noncytolytic CD8-CD4- cells that make TH2 cytokines and help B cells.. Science.

[45] Zhang L, Fung-Leung W, Miller RG (1995). Down-regulation of CD8 on mature antigen-reactive T cells as a mechanism of peripheral tolerance.. J Immunol.

[46] Park JH, Adoro S, Lucas PJ, Sarafova SD, Alag AS (2007). 'Coreceptor tuning': cytokine signals transcriptionally tailor CD8 coreceptor expression to the self-specificity of the TCR.. Nat Immunol.

[47] Cowley SC, Hamilton E, Frelinger JA, Su J, Forman J (2005). CD4-CD8- T cells control intracellular bacterial infections both in vitro and in vivo.. J Exp Med.

[48] Maldonado MA, Kakkanaiah V, MacDonald GC, Chen F, Reap EA (1999). The role of environmental antigens in the spontaneous development of autoimmunity in MRL-lpr mice.. J Immunol.

[49] Brenchley JM, Price DA, Schacker TW, Asher TE, Silvestri G (2006). Microbial translocation is a cause of systemic immune activation in chronic HIV infection.. Nat Med.

[50] Bidere N, Su HC, Lenardo MJ (2006). Genetic disorders of programmed cell death in the immune system.. Annu Rev Immunol.

[51] Bristeau-Leprince A, Mateo V, Lim A, Magerus-Chatinet A, Solary E (2008). Human TCR alpha/beta+ CD4-CD8- double-negative T cells in patients with autoimmune lymphoproliferative syndrome express restricted Vbeta TCR diversity and are clonally related to CD8+ T cells.. J Immunol.

[52] Crispin JC, Tsokos GC (2009). Human TCR-alpha beta+ CD4- CD8- T cells can derive from CD8+ T cells and display an inflammatory effector phenotype.. J Immunol.

[53] Liu YJ, Soumelis V, Watanabe N, Ito T, Wang YH (2007). TSLP: an epithelial cell cytokine that regulates T cell differentiation by conditioning dendritic cell maturation.. Annu Rev Immunol.

[54] Astrakhan A, Omori M, Nguyen T, Becker-Herman S, Iseki M (2007). Local increase in thymic stromal lymphopoietin induces systemic alterations in B cell development.. Nat Immunol.

[55] Peng SL, Madaio MP, Hayday AC, Craft J (1996). Propagation and regulation of systemic autoimmunity by gammadelta T cells.. J Immunol.

[56] Andrews BS, Eisenberg RA, Theofilopoulos AN, Izui S, Wilson CB (1978). Spontaneous murine lupus-like syndromes. Clinical and immunopathological manifestations in several strains.. J Exp Med.

